# British medical bulletin article: resourcing of palliative and end of life care in the UK during the Covid-19 pandemic

**DOI:** 10.1093/bmb/ldac013

**Published:** 2022-07-06

**Authors:** Mark Jackson

**Affiliations:** Policy & Public Affairs Manager for England, Marie Curie, England

**Keywords:** palliative care, hospice care, healthcare economics

## Abstract

**Introduction:**

Covid-19 led to a sustained increase in deaths in all four United Kingdom nations, placing strain on the UK’s palliative and end-of-life care sector and raising concerns about the long-term sustainability of the sector’s funding and resourcing model in the face of rising demand for these services in the coming decades.

**Sources of data:**

Published research, Marie Curie, King’s College London Cicely Saunders Institute, Hull York Medical School, University of Hull, University of Cambridge, National Statistics, PubMed, DOI.

**Areas of agreement:**

Care for people at the end of their lives is a core part of the UK’s health and care system with demand set to increase significantly as the UK’s population ages.

**Areas of controversy:**

The UK’s funding model for palliative and end-of-life care, with most care delivered by charitable sector providers and reliant on charitable donations, may be unsustainable in the face of increasing demand.

**Growing points:**

The Covid-19 pandemic led to rapid service innovation in palliative and end-of-life care, and providers should assess which of and how these innovations can be retained after the pandemic.

**Areas timely for developing research:**

Although there has been a rapid growth in knowledge during Covid-19, gaps still remain including: the reasons underlying shifts to deaths at home and the implications for family carers; the education needs of the wider healthcare workforce in palliative care; the impact of specialist palliative care services on the wider health system, including hospital admissions and place of death; and inequalities in the experiences of dying, death and bereavement during Covid-19 among groups such as those from lower socioeconomic groups and BAME communities.

## Introduction

The Covid-19 pandemic represents the most significant shock to the United Kingdom’s healthcare system in living memory. In addition to the exceptional pressure on frontline National Health Service (NHS) services, the pandemic led to a scale of death unprecedented in the UK in peacetime.^1^ The potential for Covid-19 to cause large numbers of deaths in the UK came to national attention on 16 March 2020, when it was suggested that, without mitigation, over 500 000 people could die from Covid-19 in the UK.^2^ Although the total number of deaths from Covid-19 by the end of 2020 was just under 90 000, all-cause mortality for the year was just over 695 000, an increase of 15% on the previous 5-year average.^3–5^

The *Better End of Life* report, a collaboration between Marie Curie, King’s College London Cicely Saunders Institute, Hull York Medical School, University of Hull and the University of Cambridge, found there was a sustained increase in deaths at home throughout 2020, with deaths at home rising as a proportion of total deaths in Great Britain (England, Wales and Scotland) from 24% to 29%, compared with the 5-year average for the same period, whereas deaths in care homes rose from 22% to 24%, with a corresponding fall in the proportion of hospital deaths from 47% to 42%.^6^ According to another study, compared with expected deaths, those who died in care homes increased by 134% in the first wave of the pandemic and 10% in the second wave, with a persistent increase in those who died at home of 67% in the first wave, which was maintained between the waves by 33%, and in the second wave by 43% above expected.^7^

This increase in mortality placed significant strain on the UK’s palliative and end-of-life care system. Note that in this article, we focus principally on services delivered by specialist palliative care providers; however, it should be noted that generalist providers such as General Practitioners and community nurses also play an important role supporting people at the end of life and did so throughout 2020.^8^

Covid-19 has therefore exposed serious vulnerabilities in the way that end-of-life care is funded and provided in the UK. Charitable hospices and other charitable providers are the main providers of this care in the UK, with only around 30% of their income coming from the UK’s governments and NHS sources.^9^ The remainder of their funding comes from charitable sources such as public giving, charitable investments and charity shop revenue. This resourcing model was placed under substantial duress during the Covid-19 pandemic, and it has raised serious questions about its long-term sustainability with demand for palliative and end-of-life care services set to rise substantially in the coming decades.

## The immediate impact of Covid-19 on palliative care resourcing

The public health response to Covid-19 had a significant and negative impact on the ability of charitable providers to raise funding to support their services. Lockdowns, social distancing and other measures to control the spread of the virus necessitated the closure of charity shops, the cancellation of large-scale events such as marathons which are major charity fundraising opportunities, street collections and other fundraising activity.^10^ Although many charities undertook emergency appeals in response to the pandemic, the impact on income generation of these measures was substantial.

Marie Curie, the largest charitable provider of palliative and end of life care services in the UK, estimated that the loss of public collections and fundraising events was $\sim$£1 million in March 2020 alone, with the closure of charity shops likely to cost $\sim$£1.5 million per month.^11^ In total, Marie Curie estimated the pandemic would lead to a fall of $\sim$25–40% in its gross income in 2020,^12^ whereas the National Council for Voluntary Organisations estimated that the wider UK charity sector would lose £4 billion in income in the first 3 months of the pandemic alone.^13^ This drop in income necessitated a number of saving and efficiency measures across the sector, such as use of the Coronavirus Job Retention Scheme (a UK Government scheme allowing employers to claim the cost of 80% of an employee’s wages if they put the employee on furlough leave due to Covid-19) and other cost-saving measures.

This fall in income came at a time when there was a sustained increase in demand for palliative and end-of-life care across the UK due to the significant increase in deaths during the pandemic. The *Better End of Life* report shows that demand for palliative care services increased across services of all types and in all parts of the UK during 2020.^14^ Although the impact of the virus itself was significant, fewer than one in seven deaths during 2020 were from Covid-19, whereas the majority were due to other causes, including long-term conditions and terminal illnesses.^15^

The combination of increased demand for services and falling income from charitable donations put patients needing palliative and end-of-life care services in the United Kingdom at substantial risk—without the provision of emergency funding from the UK’s governments it is unlikely that the sector would have been able to cope with this increased demand.

## Emergency funding from the UK’s governments

In April 2020, Her Majesty’s Treasury allocated a package of £200 million in emergency support for the charitable hospice sector in England, to ensure they remained open and continued providing services the NHS rely on them to supply, supporting the national response to the Covid-19 pandemic.^16^ A further £125 million of support for the sector in England was announced in November 2020 as part of the NHS Winter Plan.^17^ Equivalent amounts were made available in the devolved nations with funding provided by the UK Government through the Barnett formula; £27 million in Scotland, £9.3 million in Wales and £8.4 million in Northern Ireland.^18^

This emergency grant funding was used by charitable providers in the palliative and end-of-life care sector to sustain their core services through the Covid-19 pandemic, as well as enabling providers to increase the support provided to people in their own homes and provided much-needed stability throughout 2020 and into 2021.

This stability was critical. The *Better End of Life* report shows that palliative and end-of-life care services in the UK across all settings saw increased activity during 2020. Services from all UK nations and English regions reported being either slightly or a lot busier than before the Covid-19 pandemic, with between 23% and 30% of all palliative and end-of-life care services (Hands on nursing care at home/in the community; Specialist palliative home care service; Hospital palliative care advisory team; Inpatient hospice/palliative care) reporting that they were a lot busier than before.^19^

## How emergency funding was used

In many cases, this sharp increase in activity led to palliative care teams in all settings being stretched to and beyond capacity. This necessitated, and emergency funding enabled, teams to make rapid innovations, adapting their services in response to restrictions and educating, upskill and support wider health and social care professionals.^20^ Many services, for example, took on new roles in provision of education and training in symptom control for people with Covid-19 to health and social care professionals, including those in hospitals and care homes. Resources were rapidly developed to support both specialist palliative care professionals as well as wider health and social care professionals in hospital and community settings. These resources included after-death and bereavement care.

Similarly, many services increased their use of technology, including telephone and video calls. For some, this had the advantage of freeing up capacity by reducing travelling time, although services reported that remote consultations were often very time consuming due to an increase in the complexity of patient need. Services found they were providing more psychological support to people living in the community who were struggling with the impact of the pandemic.

Given the restrictions Covid-19 placed on both admissions and visitors to hospital and hospice settings, the pandemic also necessitated a shift of palliative and end-of-life care services into community settings, supporting people in their homes and in care homes. Some inpatient hospice units reported a drop in inpatient referrals, whereas others needed to close beds due to guidance on infection control. This often prompted a shift in resources from inpatient to community provision where needs were greatest.

This shift to supporting people at home or elsewhere in the community was vital given the impact Covid-19 had on where people died in 2020, with more people dying at home and in the community. Although the initial shift in place of death was doubtlessly driven by Covid-19 and peaked during the first wave of the pandemic, this increase was sustained throughout 2020 even outside of Covid-19 waves.^21^

## Resourcing beyond funding

It is important to underline that the resourcing of palliative and end-of-life care services depends on more than how these services are funded. As the *Better End of Life* report shows, the challenges faced by services throughout the pandemic went beyond falls in income and disruption to funding. Although the likelihood that many people would die as a result of Covid-19 was recognized from the early stages of the pandemic, there was little focus from the UK’s healthcare system on the need for palliative and end-of-life care or recognition of palliative care as an essential part of the Covid-19 response.^22^

This led to palliative and end-of-life care services not being seen as ‘frontline NHS’ services throughout the initial stages of the pandemic, which led to resources being hard to secure regardless of the availability of funds. The *Better End of Life* report found that shortages of Personal Protective Equipment (PPE) were reported by between 33%and 61% of services in all parts of the UK, most commonly in the North West of England and West Midlands.^23^ PPE shortages included masks, aprons and face shields, as well as other essential equipment, including cleaning products, waste disposal products and body bags.

Similarly, services reported shortages of other equipment—most commonly syringe pumps and the associated lines/needles needed—and essential medications for symptom and pain control were reported by significant minorities of services in the UK, with as many as 35% of services reporting equipment shortages and 42% of services reporting shortages of medication in some parts of the country.^24^ Procuring adequate supplies of PPE, equipment and medication was affected by the perception that services were not ‘frontline NHS’ services and was therefore extremely time consuming in the initial weeks of the pandemic until procurement challenges were rectified, and reduced the ability of services to provide direct patient care in this early period of the crisis.

The palliative and end-of-life care sector also saw significant disruption due to staff shortages throughout the pandemic. These were most common in Wales and London, where 60% of services reported having experienced staff shortages throughout 2020.^25^ These shortages were as a result of staff sickness, in addition to staff having to shield or self-isolate in line with government public health guidance. This was compounded by barriers in access to staff testing, again caused by palliative and end-of-life care staff working for independent and charitable providers not being seen as ‘frontline NHS.’ In addition, in some cases palliative and end-of-life care staff were redeployed from palliative care into other roles due to staffing pressures elsewhere in the NHS caused by the pandemic.

## The long-term sustainability of palliative and end-of-life care in the UK

Emergency funding provided by the UK’s governments was critical in supporting the palliative and end-of-life care sector to cope with the increased demand and significant resource pressures brought on by Covid-19. This funding helped services to absorb the impact of a substantial fall in charitable donations, enabled palliative and end-of-life care teams to cope with increased demand, shift resources to community settings and rapidly adapt their services, and mitigate the impact of shortages in staff, PPE, medicines and equipment throughout 2020.^26^

However, this funding was always intended to be a short-term expedient to address the immediate impact of the Covid-19 crisis on the palliative and end-of-life care sector, and not a long-term solution. The broader challenges facing palliative and end-of-life care in the UK are long-term and systemic and will not be addressed by short-term injections of emergency funding. As a result of the UK’s ageing population, by 2040 it is estimated that the number of people aged over 85 will double to >3.2 million^27^ and 100 000 more people will be dying in the UK each and every year.^28^ The scale of mortality experienced in 2020 should therefore be seen not as a one-off event but as a precursor to what will become the ‘new normal’ in the coming decades.

Many of these older people will be living with multiple, complex conditions toward the end of their lives and it is likely that the majority of the additional people dying each year would benefit from palliative and end-of-life care. Estimates suggest that between 75% and 90% of all people who die in the UK would benefit from palliative care,^29^ underlining the significant and sustained increase in demand for these services that will take place over the coming decades.

The difficulties faced by the palliative and end-of-life care sector in 2020 over the course of the pandemic demonstrate that there is a significant risk that the sector will be unable to cope with this rising demand under the current model for delivering palliative and end-of-life care in the UK. Already, as many as 50% of people who may benefit from palliative and end-of-life care do not receive it,^30^ with particular inequities in access to this care being experienced by Black, Asian and Ethnic Minority (BAME) communities, people living in more deprived areas, people who do not live with a spouse or partner and people with non-cancer conditions.^31^

These inequalities reflect long-standing health inequalities in the UK^32^ and were also reflected in the disproportionate impact of Covid-19 on people living in more deprived areas and BAME communities.^33^ As demand for palliative and end-of-life care services increases in the future, there is a significant risk that these inequalities in access will widen under the sector’s current funding model. Addressing the way that palliative and end-of-life care services are funded and delivered in the UK must be an urgent priority if these vital services are to be available to everybody who needs them in future.

## What is needed in future?

New models for delivering palliative and end-of-life care in the community will be needed in the UK, to reduce pressures on the NHS and fulfil patient preferences for dying at home. Larger numbers of family members and carers will require support through dying, death and bereavement. Improving the resourcing of services in the community is of particular importance given the rising numbers of people in the UK who die at home, an ongoing trend that was accelerated by the Covid-19 pandemic in 2020.^34^

The flexibility demonstrated by palliative care services during the pandemic, with palliative care teams shifting services and activities into community settings,^35^ is a strong example of the increased agility that service models may need in future, with staff able to provide care flexibly across care settings in the community as and where people at the end of life need care. Experience in Wales during the pandemic, for example, has demonstrated good practice in this area, with some teams shifting care from one setting to another—for example in response to a Covid-19 outbreak reducing capacity in inpatient settings—and staff being redeployed to another setting or supporting other colleagues.^36^^,^^37^

The Covid-19 pandemic has also underlined the need for a long-term, sustainable funding solution for palliative and end-of-life care. A funding model that relies so heavily on the generosity of the public is unlikely to be sustainable in the face of a sustained increase in demand for services, and there is a serious risk that the sector could collapse under the weight of the growing needs of the population and financial instability.

A new funding settlement that recognizes care for people at the end of their lives as a core part of the health and care system, with statutory sources of funding making up a larger proportion of the sector’s funding, is urgently needed. Requiring local commissioners such as Integrated Commissioning Systems (ICS) in England to commission specialist palliative care services in all parts of the country would help ensure that commissioned services are available in all areas and ensure a greater proportion of the sector’s funding comes from statutory sources. However, the Health & Care Bill currently being debated by the UK Parliament does not require these services to be commissioned by ICS Boards.

Many of these challenges reflect wider issues in the UK’s health and social care system and addressing these will also require addressing those challenges. Ensuring that more people are supported to be cared for at home or in the community at the end of their lives, for example, will require addressing both the inconsistent delivery of Fast Track Continuing Healthcare (funding which enables people to be cared for outside of hospital at the end of their lives) and capacity gaps in the social care system that often mean people near the end of life are unable to leave hospital or otherwise cannot stay at home.^38^ Similarly, action to address workforce gaps in the wider health and social care system, which already number >100 000 in the NHS and 122 000 in adult social care^39^ and which the King’s Fund warns could reach 250 000 in the NHS alone by 2030,^40^ will be critical if the UK’s health and care system is to cope with the increasing demand for care for people at all stages of life, especially those nearing the end of their lives.

## Data availability statement

The data that support the findings of this article are available from the corresponding author, Mark Jackson, on reasonable request.
